# Epidemiological characteristics of obstructive sleep apnea in a hospital-based historical cohort in Lebanon

**DOI:** 10.1371/journal.pone.0231528

**Published:** 2020-05-15

**Authors:** Marie-Louise Coussa-Koniski, Elie Saliba, Francine K. Welty, Mary Deeb

**Affiliations:** 1 Respiratory Division, Lebanese American University Medical Center-Rizk Hospital (LAUMC-RH), Beirut, Lebanon; 2 Lebanese American University, Gilbert and Rose-Marie Chagoury School of Medicine, Byblos, Lebanon; 3 Beth Israel Deaconess Medical Center, Harvard Medical School, Boston, Massachusetts, United States of America; Weill Cornell Medical College in Qatar, QATAR

## Abstract

The objective of our study was to characterize and analyze the associations between OSA (obstructive sleep apnea) and other clinical variables in adult patients referred for sleep evaluation by polysomnography at a referral center in Beirut, Lebanon, in terms of sociodemographic features, symptoms presentation and comorbidities, and evaluate the burden of comorbidities associated with this disease. All individuals with suspected Sleep Apnea referred (January 2010-September 2017) for a one-night polysomnography were included. Demographics, self-reported symptoms and comorbidities were documented. The relationship between OSA severity and the presence of symptoms and comorbidities were evaluated using multivariate logistic regression. Overall, 663 subjects were assessed. Of these, 57.3% were referred from chest physicians, and sleep test results were abnormal in 589 subjects (88.8%) of whom 526 patients (89.3%) fulfilled diagnostic criteria for OSA; 76.3% were men and women were on average older. OSA was severe in 43.2% and more severe in men. Almost all patients were symptomatic with ~2–4 symptoms per patient and women presented with symptoms that are more atypical. Comorbidities were significantly higher in women. In the multivariate analysis, age, male sex, obesity, symptoms of snoring, excessive daytime somnolence and witnessed apneas were associated with OSA severity. Only age and obesity were associated with self-reported diagnosis of hypertension and diabetes. This is the first study in Lebanon to explore the characteristics of patients with polysomnography-diagnosed OSA. High prevalence of severe OSA and low referral rates in the medical community support promoting awareness for an earlier diagnosis and more personalized approach in this country.

## Introduction

Obstructive Sleep Apnea (OSA) is a common, treatable sleep disorder characterized by recurrent episodes of complete (apnea) or partial (hypopnea) collapse of the upper airway that impairs normal ventilation during sleep, causing intermittent hypoxemia, microarousals, increased oxidative stress, inflammation and sleep fragmentation which are important mediators of metabolic, cardiovascular and neurocognitive risk [1]. OSA is well characterized as an independent risk factor for hypertension, stroke, heart failure, atrial fibrillation, coronary heart disease and diabetes, as well as of cardiovascular mortality [2]. In addition, a possible association of OSA with cancer incidence and mortality has been suggested recently [3]. There is evidence from studies in humans, as well as in animals, for a role of OSA as a potential risk factor for a more aggressive course or presentation of cancer [3–7]. However, the nature of any such association between OSA and cancer remains unclear [3].

Obstructive sleep apnea is perhaps one of the most common respiratory disorders and has become a major public health problem. Estimates of the prevalence of OSA in the general population vary significantly based on the source population and the definition of OSA used, with respect to the testing methodology, scoring algorithm [8–13], and diagnostic criteria, which were updated in 2012 [14]. A more recent study using current diagnostic definitions [9, 13] reported a substantially higher prevalence of moderate to severe OSA of 23% in women and 49% in men.

Following the demonstration of the strong association between OSA and cardiovascular disease (11), awareness of OSA has grown in North America and Western Europe over the last thirty years as a result of public awareness policies. For example, consultations for OSA in the United States rose over tenfold during the 1990s [15]. In other parts of the world, awareness of OSA may be much lower and information on the epidemiology and management of this condition are scarce. In the Middle East, it is particularly important to document OSA due to the high prevalence of obesity, diabetes and smoking in this region, obesity being one of the major risk factors for OSA [1, 16]. According to data from the 2013 Global Burden of Disease Study, 20.3% of men and 33.9% of women in the region are obese, and diabetes affects around 15% of the Lebanese population [17]. Some data on the prevalence of OSA in the region are available for Jordan, Saudi Arabia and the United Arab Emirates [18] but these studies are limited to certain categories of patients and showed that the risk of OSA evaluated from symptoms reported by patients attending primary care clinics, ranged from 16.8% in Jordan to 33.0% in Saudi Arabia [19–22].

The objective of our study was to characterize, analyze and compare the associations between OSA and other clinical variables in a cohort of adult patients referred for sleep evaluation by polysomnography at a referral center in Beirut, Lebanon, in terms of sociodemographic features, symptom presentation and comorbidities.

## Methods

This study was a retrospective review of data of adult outpatients referred for suspected sleep apnea to the sleep laboratory of the Lebanese American University Medical Centre-Rizk Hospital (LAUMC-RH) in Beirut, Lebanon, over a period from January 2010 to September 2017.

### Polysomnography

One night polysomnography was performed using an Alice 6 (17 channels) Philips, Respironics. The following variables were monitored: electroencephalogram (6 channels), electro-oculogram (2 channels), electromyogram (4 channels: 2 submental and 2 anterior tibialis muscles), electrocardiogram (one channel D2 modified), snoring, and body position. The airflow was monitored using a thermocouple and a nasal pressure transducer (Pro-Tech Services Inc., Mukilteo, Washington). Chest and abdominal respiratory inductance plethysmography monitored the respiratory effort of each patient. Arterial oxygen saturation (SpO_2_) and pulse were recorded using a pulse oximeter (Nonin, model 9500, Plymouth, USA). An electrocardiogram was recorded continuously.

All polysomnographies were performed and scored by an experienced sleep technician using the American Academy of Sleep Medicine (AASM) guidelines for sleep studies [14, 23]. For the patients evaluated from January 2010 until October 2012, hypopnea episodes were identified according to the “recommended: 4A” or “alternative: 4B” definitions of hypopnea in the American Academy of Sleep Medicine 2007 scoring manual [23]. Hypopnea was identified EITHER “if all the following criteria are met: the nasal pressure signal excursions (or those of the alternative hypopnea sensor) drop by ≥30% of baseline, for a period lasting at least 10 seconds with ≥4% desaturation from pre-event baseline and at least 90% of the event`s duration met the amplitude reduction of criteria for hypopnea” as per “the recommended”hypopnea definition, OR “if all the following criteria are met: the nasal pressure signal excursions (or those of the alternative hypopnea sensor) drop by ≥50% of baseline, for a period lasting at least 10 seconds with ≥3% desaturation from pre-event baseline or the event is associated with arousal and at least 90% of the event`s duration met the amplitude reduction of criteria for hypopnea” as per “the alternative” hypopnea definition. For the patients diagnosed since updated rules for scoring respiratory events in sleep were published in 2012 [14], hypopnea was identified “if all the following are met: the peak signal excursions drop by ≥30% of pre-event baseline using nasal pressure or an alternative hypopnea sensor lasting ≥10 seconds and if there is ≥3% oxygen desaturation from pre-event baseline or the event is associated with an arousal”.

The apnea hypopnea index (AHI) was calculated as the combined number of apnea and hypopnea episodes per hour sleep. Patients with an AHI≥5 combined with clinical symptoms or AHI≥15 without symptoms were considered to have OSA. The severity of sleep apnea was assessed using the Apnea-Hypopnea Index (AHI) as recommended in the AASM practice guidelines [23]. Sleep apnea/hypopnea was considered mild if the AHI was 5–15, moderate if the AHI was 15–30 and severe if the AHI was >30. Each test result was reviewed and the diagnosis confirmed by a sleep medicine physician.

### Patient evaluation

Information on sleep symptoms and other medical conditions was also collected at the time of the visit to the sleep laboratory during a direct interview by a physician who took a complete history. In addition, the patient completed a questionnaire about sleep symptoms. Anthropometric and demographic measurements for each subject included weight in kilograms, height in meters, neck circumference in centimeters measured at the level of the cricothyroid membrane, blood pressure, pulse, and body mass index (BMI) and were documented upon arrival at the sleep laboratory.

BMI (in Kg/m^2^) was categorized using the WHO and NIH classification into normal: 18.5–24.9, overweight: 25–29.9 Kg/m^2^, obese 30–39.9 Kg/m^2^ and morbid obese ≥40 Kg/m^2^. Comorbidities were documented from patient interview, taking into account clinical history, diagnoses made by their treating physician and treatments prescribed. Subjective excessive daytime somnolence (EDS) was evaluated using the Epworth Sleepiness Score (ESS) [24]. An ESS>10 was defined as subjective EDS. Nocturia was identified from the patient as “the complaint” of having to wake at night one or more times to void in accordance with the International Continence Society definition in 2002 [25].

### Data collection

At the time of the study (2017), all the medical records of patients referred for a diagnosis of OSA that were available since 2010 were retrieved and reviewed for completeness of data by the principal investigator. A standard data sheet was designed to transcribe the data to be extracted from the patient records. Information was extracted on age, gender, weight, height, neck circumference, smoking status (current, former or never), symptoms (snoring, nocturnal choking, witnessed apnea, excessive daytime sleepiness, morning headache, dizziness, syncope, leg discomfort at night, nocturia and insomnia), comorbidities (coronary artery disease, myocardial infarction, stroke, arrhythmias, chronic heart failure, hypertension, asthma, chronic obstructive pulmonary disease, diabetes, dyslipidemia, gastroesophageal reflux disorder, rhino sinusitis, dysthyroidism, kidney disease, musculoskeletal disorders and depression). A medical resident at LAUMC-RH performed the data extraction in the sleep laboratory offices. All the polysomnography records were reviewed by a sleep specialist. All the polysomnography records were reviewed by a sleep specialist to ensure that the original apnea/hypnea criteria were fulfilled.

### Statistical analysis

Data presentation is principally descriptive and no *a priori* hypotheses were tested. Continuous data are presented as mean values with standard deviation (SD) and categorical values as frequency counts and percentages. Missing data were not replaced. Variables were compared between men and women and across OSA severity grades using the χ^2^ test for categorical variables and the Wilcoxon test for continuous values. Two-sided tests were used in all cases and a probability threshold of 0.05 was considered significant. Variables associated with severe OSA, hypertension and diabetes, as dependent variables were investigated using multivariate logistic regression analysis. In a first step, all documented variables were evaluated independently in a univariate analysis. Those variables for which a significant association (*p* <0.05) with the dependent variable was observed were then entered into a multivariate analysis and odds ratios generated. Age, sex and BMI were included in all the multivariate analyses. All data analysis was performed using IBM, SPSS statistical software Version 25.

### Ethical considerations

The study was conducted according to the principles of Good Epidemiological Practice and pertinent international and Lebanese regulations for medical research. The study was reviewed and approved by the Institutional Board Ethical Committee of the Lebanese American University (LAU—registration number: UMCRH.MC1.28/June/2012). Since this was a retrospective chart review, patients did not provide informed consent. However, access to the data was completely anonymised, by hiding the name of the patient from their medical records before the study started. The IRB committee waived the requirement for informed consent.

## Results

### Patients

**Fig 1:**
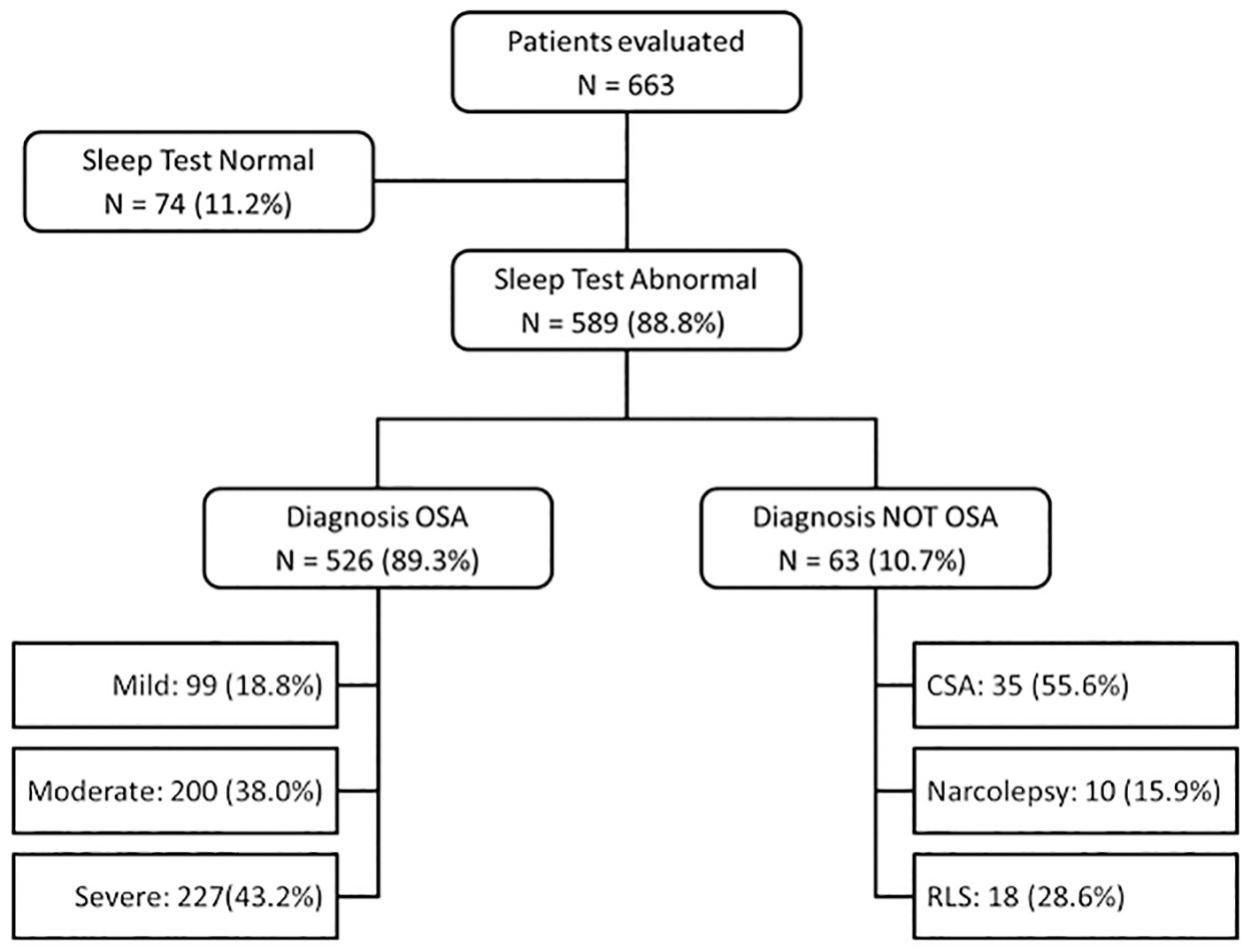
Distribution of patients by diagnosis. Percentages are calculated with respect to the mother cell in each case. CSA: central sleep apnea; OSA: obstructive sleep apnea; RLS: restless legs syndrome.

Over the seven-year evaluation period, 663 patients were referred to the sleep laboratory with suspected sleep-disordered breathing. The majority of referrals were from chest physicians (57.3%) with only 4.6% referred by a general practitioner and 10.0% consulted spontaneously without a referral from a physician. The remaining referrals were from other specialists (cardiologists, ENT specialists, neurologists or endocrinologists). The sleep test results were abnormal in 589 subjects (88.8%). The majority of patients with abnormal sleep test results fulfilled diagnostic criteria for OSA (526 patients; 89.3%). The distribution of patients by diagnosis is illustrated in Fig 1.

The characteristics of the study population are presented in Table 1. Overall, 76.3% of patients were men and the mean age was 60.8±16 years, with women being on average older than men (p = 0.005). The proportion of patients obese or morbidly obese was 66%, the distribution of BMI classes differing significantly between men and women. The proportion of women morbidly obese (29.8%) was higher than the male proportion (10.4%). The proportion of male patients who were current smokers (48.1%) was significantly (p = 0.012) higher than among women (33.9%) who were current smokers.

**Table 1:** Patient characteristics.

	**Men**	**Women**	**Total**	***p***
	N = 506	N = 157	N = 663	
**Age Group**	*N = 506*	*N = 157*	*N = 663*	
20–50	133 (26.4%)	34 (21.3%)	167 (25.3%)	**0.002**
50–60	123 (24.3%)	24 (15.5%)	147 (22.3%)	
60–70	106 (20.7%)	30 (19.4%)	136 (20.5%)	
>70	144 (28.6%)	69 (43.9%)	213 (32.0%)	
**Age (Years)**				
Mean ± SD	59.8 ± 15.7	63.9 ± 16.6	60.8 ± 16.0	**0.005**
**Neck circumference**	*N = 377*	*N = 118*	*N = 495*	
Mean ± SD	42.9 ± 3.5	38.0 ± 5.1	41.8 ± 4.6	**0.020**
**BMI**	*N = 480*	*N = 151*	*N = 631*	
Normal	46 (9.6%)	28 (18.5%)	74 (11.7%)	**<0.001**
Overweight	154 (32.1%)	23 (15.2%)	177 (28.1%)	
Obese	230 (47.9%)	55 (36.4%)	285 (45.2%)	
Morbid Obese	50 (10.4%)	45 (29.8%)	95 (15.1%)	
**Smoking**	*N = 412*	*N = 115*	*N = 527*	
Current	198 (48.1%)	39 (33.9%)	237 (45.0%)	**0.012**
Former	104 (25.2%)	31 (27.0%)	135 (25.6%)	
Never	110 (26.7%)	45 (39.1%)	155 (29.4%)	
**Symptoms**				
Snoring	389 (77.2%)	89 (56.7%)	478 (72.3%)	**<0.001**
Nocturnal choking	210 (41.7%)	45 (28.7%)	255 (38.6%)	**0.003**
Witnessed apnea	74 (14.7%)	17 (11.0%)	91 (13.9%)	0.235
Excessive daytime sleepiness	352 (70.2%)	111 (70.7%)	463 (70.3%)	0.889
Morning headache	101 (20.2%)	48 (30.6%)	149 (22.6%)	**0.007**
Dizziness	59 (11.7%)	25 (15.9%)	84 (12.7%)	0.169
Syncope	34 (6.8%)	11 (7.1%)	45 (6.8%)	0.884
Leg discomfort at night	53 (10.6%)	34 (21.7%)	87 (13.2%)	**<0.001**
Nocturia	71 (14.1%)	28 (17.8%)	99 (15.0%)	0.259
Insomnia	129 (25.4%)	29 (18.5%)	157 (23.8%)	0.075
	**Men**	**Women**	**Total**	***p***
**Comorbidities**	N = 506	N = 157	N = 663	
*Cardiovascular*				
Coronary artery disease	45 (9.0%)	20 (12.7%)	65 (9.9%)	**0.001**
Myocardial infarction	4 (0.8%)	2 (1.3%)	6 (0.9%)	0.579
Stroke	10 (2.0%)	2 (1.3%)	12 (1.8%)	0.568
Arrhythmias	50 (9.9%)	16 (10.3%)	66 (10.0%)	0.890
Chronic heart failure	18 (3.6%)	16 (10.3%)	34 (5.2%)	**0.001**
Hypertension	243 (48.4%)	81 (52.3%)	324 (49.3%)	0.402
*Respiratory*				
Asthma	41 (8.2%)	11 (7.0%)	52 (7.9%)	0.633
Chronic obstructive pulmonary disease	39 (7.8%)	30 (19.2%)	69 (10.5%)	**<0.001**
*Metabolic Disease*				
Diabetes	82 (16.3%)	45 (28.8%)	127 (19.3%)	**0.001**
Dyslipidemia	216 (43.1%)	64 (41.0%)	280 (42.6%)	0.645
*Other co-morbidities*				
Gastroesophageal reflux disorder	223 (45.1%)	58 (37.2%)	281 (43.2%)	0.083
Rhino sinusitis	131 (26.1%)	32 (20.5%)	163 (24.8%)	0.155
Dysthyroidism	30 (6.0%)	41 (26.1%)	71 (10.8%)	**<0.001**
Kidney Disease	29 (5.8%)	7 (4.5%)	36 (5.5%)	0.530
Musculoskeletal disorders	37 (7.4%)	28 (17.8%)	65 (9.9%)	**<0.001**
Depression	40 (8.0%)	29 (18.5%)	69 (10.5%)	**<0.001**

Almost all patients were symptomatic (97%) with an average of 2–4 symptoms per patient. The mean number of symptoms did not differ (p = 0.347) between women (2.8 ± 1.5) and men (2.9 ± 1.5). The most frequently reported symptoms were snoring (72.3%) and EDS (70.3%). Snoring and nocturnal choking were significantly more frequent in men and morning headache and leg discomfort at night significantly more frequent in women.

Comorbidities were reported by 90% of the referred patients with 60% reporting 2–4 comorbidities. The mean number of comorbidities was significantly higher (p <0.001) in women (3.1 ± 1.8) than in men (2.4 ± 1.7). The most common comorbidities reported were hypertension (49.3%), dyslipidemia (42.6%) and gastro-esophageal reflux disorder (GERD; 43.2%). Diabetes was reported by 19.3% of the subjects. Coronary artery disease, chronic heart failure, chronic obstructive pulmonary disease (COPD), diabetes, dysthyroidism, musculoskeletal disorders and depression were all significantly more frequent in women.

#### Variables related to the severity of obstructive sleep apnea

**Fig 2:**
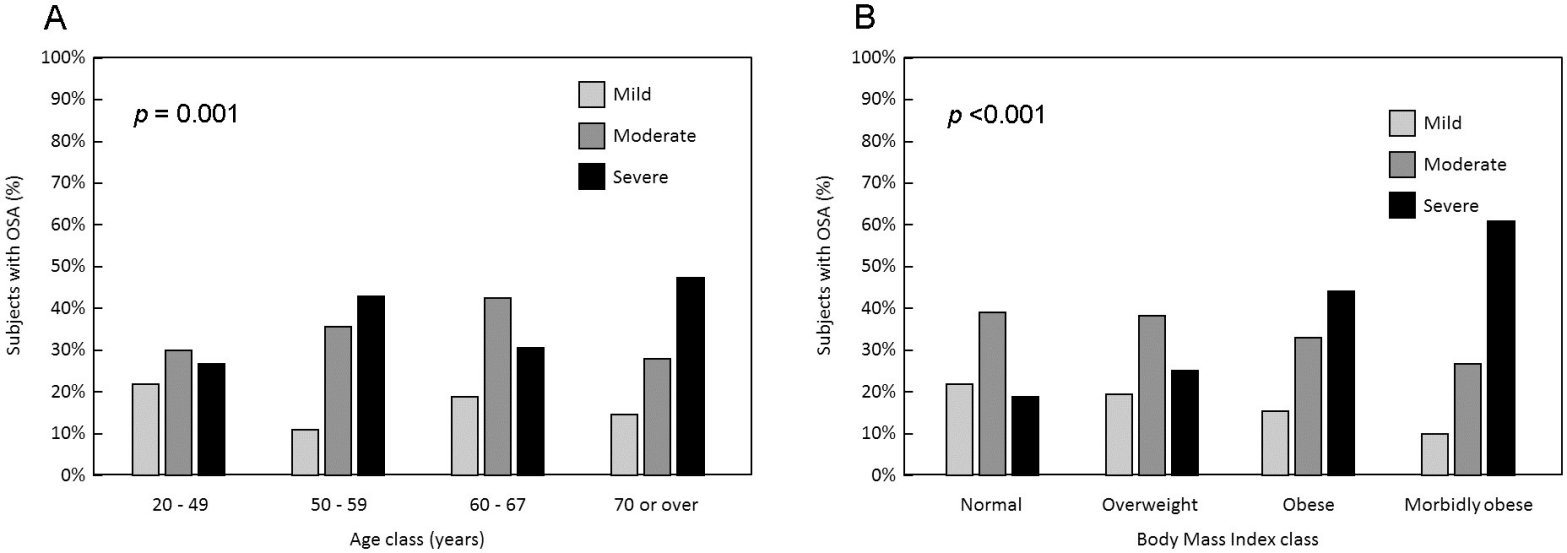
Relationship between AHI severity grade and age (A) and body mass index (B).

**Fig 3:**
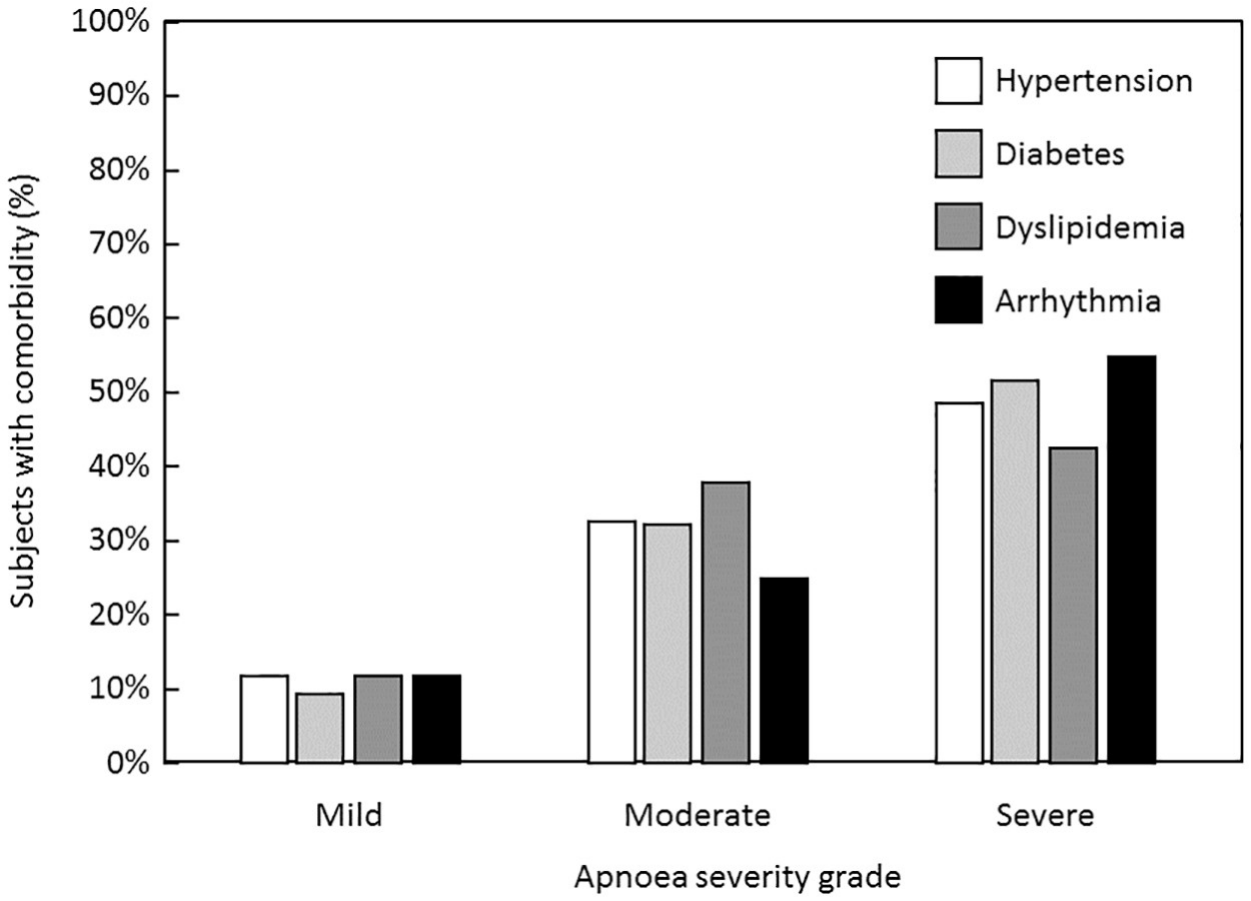
Distribution of AHI severity grade in selected comorbidities.

The distribution of patient variables as a function of apnea severity is presented in Table 2. The mean AHI was 32.6 ± 27.0 and was ≥30 in 227 subjects (43.2%) with obstructive sleep apnea. OSA was significantly more frequently severe in men (46.6%) than in women (30.4%) and the distribution of severity grades differed significantly between men and women (*p* <0.004). Severity of sleep apnea also increased with age and BMI class (Fig 2A and 2B). A general trend was observed for symptoms to be more frequent in more severe disease, but this relationship was only significant for snoring, nocturnal choking, nocturia, EDS and witnessed apnea. Arrhythmias, hypertension, diabetes and dyslipidemia were more frequent in more severe sleep apnea (Fig 3). None of the other documented comorbidities listed in Table 2 were associated with the severity of sleep apnea.

**Table 2:** Variables related to the severity of sleep apnea/hypopnea.

	Obstructive sleep apnea			Total N = 526	*p*
	Mild (AHI: 5-<15) N = 99	Moderate (AHI: 15-<30) N = 200	Severe (AHI: ≥30) N = 227		
**Sex**					
Male	69 (16.7%)	152 (36.7%)	193 (46.6%)	414	**0.004**
Female	30 (26.8%)	48 (42.9%)	34 (30.4%)	112	
**Age Group**					
20–50	32 (27.8%)	44 (38.3%)	39 (33.9%)	115	**0.001**
50–60	15 (12.2%)	49 (39.8%)	59 (48.0%)	123	
60–70	24 (20.5%)	54 (46.2%)	39 (33.3%)	117	
≥70	28 (16.4%)	53 (31.0%)	90 (52.6%)	171	
**Age (Years)**					
Mean ± SD	59.1±16.3	60.2±14.9	63.8±14.2		**0.008**
**BMI**					
Normal	14 (27.5%)	25 (49.0%)	12 (23.5%)	51	**<0.001**
Overweight	31 (23.5%)	61 (46.2%)	40 (30.3%)	132	
Obese	40 (16.6%)	86 (35.7%)	115 (47.7%)	241	
Morbid Obese	9 (10.2%)	24 (27.3%)	55 (62.5%)	88	
**Symptoms**					
Snoring	65 (16.0%)	151 (37.2%)	190 (46.8%)	406	**0.002**
Nocturnal chocking	31 (13.4%)	86 (37.2%)	114 (49.4%)	231	**0.009**
Leg discomfort at night	16 (23.5%)	27 (39.7%)	25 (36.8%)	68	0.398
Nocturia	9 (10.0%)	27 (30.0%)	54 (60.0%)	90	**0.001**
Insomnia	24 (19.4%)	45 (38.2%)	55 (44.4%)	124	0.893
Dizziness	12 (24.1%)	24 (34.8%)	33 (47.8%)	69	0.717
Syncope	5 (15.2%)	13 (39.4%)	15 (45.4%)	33	0.872
Morning headache	18 (15.1%)	46 (38.7%)	55 (46.2%)	119	0.548
Excessive daytime sleepiness	65 (17.4%)	125 (33.5%)	183 (49.1%)	373	**<0.001**
Witnessed apnea	9 (11.4%)	28 (35.4%)	42 (53.2%)	79	0.038
**Comorbidities**					
Cardiovascular					
Coronary artery disease	8 (15.1%)	21 (39.6%)	24 (45.3%)	53	0.778
Myocardial infarction	1 (20.0%)	1 (20.0%)	3 (60.0%)	5	0.682
Stroke	2 (20.0%)	2 (20.0%)	6 (60.0%)	10	0.465
Arrhythmias	7 (12.7%)	15 (27.3%)	33 (60.0%)	55	**0.032**
Chronic heart failure	6 (22.2%)	9 (33.3%)	12 (44.4%)	27	0.825
Hypertension	35 (12.8%)	96 (35.0%)	143 (52.2%)	274	**<0.001**
Respiratory					
Asthma	9 (22.0%)	15 (36.6%)	17 (41.5%)	41	0.863
Chronic obstructive pulmonary disease	11 (20.0%)	22 (40.0%)	22 (40.0%)	55	0.863
Metabolic Disease					
Diabetes	11 (10.0%)	38 (34.5%)	61 (55.5%)	110	**0.006**
Dyslipidemia	31 (12.8%)	100 (41.2%)	112 (46.1%)	243	**0.008**
Other co-morbidities					
Gastroesophageal reflux disorder	42 (18.7%)	82 (36.4%)	101 (44.9%)	225	0.820
Rhino sinusitis	27 (20.3%)	53 (39.8%)	53 (39.8%)	133	0.624
Dysthyroidism	14 (24.6%)	16 (28.1%)	27 (47.4%)	57	0.220
Kidney disease	6 (18.2%)	12 (36.4%)	15 (45.5%)	33	0.970
Musculoskeletal disorders	12 (31.6%)	13 (34.2%)	13 (34.2%)	38	0.103
Depression	10 (20.4%)	17 (34.7%)	22 (44.9%)	49	0.875

### Variables related to excessive daytime sleepiness

Although EDS was a highly prevalent complaint, no significant difference was observed in the prevalence of EDS between sex and age groups, while obesity was significantly related to the prevalence of EDS. The only symptoms related significantly to EDS were snoring, leg discomfort at night and insomnia. Arrhythmias and hypertension were the only reported co-morbidities associated with EDS. The distribution of patient variables as a function of EDS is presented in Table 3.

**Table 3:** Variables related to the presence of excessive daytime sleepiness.

	Excessive daytime sleepiness		Total	*p*
	Yes	No		
**Sex**	*N = 373*	*N = 150*	*N = 523*	
Male	291 (70.8%)	120 (29.2%)	*411*	0.617
Female	82 (73.2%)	30 (26.8%)	*112*	
**Age Group**				
20–50	82 (72.6%)	31 (27.4%)	113	0.573
50–60	87 (70.7%)	36 (29.3%)	123	
60–70	78 (66.7%)	39 (33.3%)	117	
70 +	126 (74.1%)	44 (25.9%)	170	
**Age (Years)**				
Mean ± SD	61.9 ± 15.1	60.8 ± 14.9	60.8 ± 16.0	0.470
**BMI**	*N = 366*	*N = 143*	*N = 509*	
Normal	29 (56.9%)	22 (43.1%)	51	**<0.001**
Overweight	80 (61.1%)	51 (38.9%)	131	
Obese	181 (75.7%)	58 (24.3%)	239	
Morbid Obese	76 (86.4%)	12 (13.6%)	88	
Symptoms*				
Snoring	300 (74.3%)	104 (25.7%)	404	**0.006**
Nocturnal choking	161 (70.3%)	68 (29.7%)	229	0.669
Witnessed apnea	51 (64.6%)	28 (35.4%)	79	0.153
Morning headache	85 (71.4%)	34 (28.6%)	119	0.171
Dizziness	54 (78.3%)	15 (21.7%)	69	0.169
Syncope	24 (72.7%)	9 (27.3%)	33	0.842
Leg discomfort at night	58 (85.3%)	10 (14.7%)	68	**0.006**
Nocturia	71 (78.9%)	19 (21.1%)	90	0.081
Insomnia	99 (80.5%)	24 (19.5%)	123	**0.010**
	**Yes**	**NO**	**Total**	***p***
Comorbidities*	N = 373	N = 150	N = 523	
*Cardiovascular*				
Coronary artery disease	39 (73.6%)	14 (26.4%)	53	0.683
Myocardial infarction	3 (60.0%)	2 (40.0%)	5	0.568
Stroke	7 (70.0%)	3 (30.0%)	10	0.913
Arrhythmias	48 (87.3%)	7 (12.7%)	55	**0.006**
Chronic heart failure	19 (70.4%)	8 (29.6%)	27	0.895
Hypertension	206 (75.5%)	67 (24.5%)	273	**0.044**
*Respiratory*				
Asthma	34 (82.9%)	7 (17.1%)	41	0.087
Chronic obstructive pulmonary disease	45 (81.8%)	10 (18.2%)	55	0.075
*Metabolic Disease*				
Diabetes	80 (72.7%)	30 (27.3%)	110	0.756
Dyslipidemia	174 (71.6%)	69 (28.4%)	243	0.954
*Other co-morbidities*				
Gastroesophageal reflux disorder	164 (73.5%)	59 (26.5%)	223	0.306
Rhino sinusitis	93 (70.5%)	39 (29.5%)	132	0.762
Dysthyroidism	43 (75.4%)	14 (24.6%)	57	0.469
Kidney Disease	25 (75.8%)	8 (24.2%)	33	0.569
Musculoskeletal disorders	30 (78.9%)	8 (21.1%)	38	0.274
Depression	39 (79.6%)	10 (20.4%)	49	0.177

### Multivariate analysis of factors associated with the severity of obstructive sleep apnea

The multivariate analysis, performed in the 526 patients with a diagnosis of OSA, evaluated variables associated with severe as opposed to mild/moderate OSA. In this multivariate model (Table 4), increasing age, male sex and obesity were associated with severe OSA compared to mild/moderate AHI. The only symptoms to be associated with severe OSA were nocturia (odds ratio: 2.35 [95% CI: 1.39–3.98]), EDS (odds ratio: 1.89 [1.19–2.99]), and witnessed apnea (odds ratio: 1.77 [1.02–3.07]). After adjustment for age, sex and BMI, none of the comorbidities were significantly associated with severe OSA. However, hypertension had a borderline significance with a p-value of 0.084.

**Table 4:** Variables associated with severity of obstructive sleep apnea: Multiple logistic regression analysis.

Variable	Reference	Univariate *p*	Multivariate *p*	Multivariate OR [95% CI]
Age	≥70 years vs <70 years	0.001	0.005	**1.91 [1.22–3.00]**
Sex	Male vs female	0.002	<0.001	**3.16 [1.86–5.39]**
BMI	≥30 kg/m^2^ vs <30 kg/m^2^	<0.001	<0.001	**2.39 [1.53–3.73]**
Snoring	Present vs absent	0.001	0.091	1.57 [0.93–2.63]
Nocturnal choking	Present vs absent	0.013	0.180	1.32 [0.88–1.98]
Witnessed apnea	Present vs absent	0.038	0.044	**1.77 [1.02–3.07]**
EDS	Present vs absent	<0.001	0.007	**1.89 [1.19–2.99]**
Nocturia	Present vs absent	<0.001	0.002	**2.35 [1.39–3.98]**
Hypertension	Present vs absent	<0.001	0.084	1.45 [0.95–2.22]
Diabetes	Present vs absent	0.004	0.352	1.29 [0.75–2.22]
Dyslipidemia	Present vs absent	0.040	0.703	1.09 [0.72–1.65]
Arrhythmia	Present vs absent	0.009	0.348	1.59 [0.82–3.06]

BMI: body mass index; EDS: excessive daytime sleepiness

### Variables related to comorbid hypertension and diabetes

In the multivariate models (S1 Table) increasing age and morbid obesity, but not sex, were associated with a known diagnosis of hypertension. Neither the severity of OSA nor any of the sleep apnea symptoms identified in univariate analysis remained significantly associated with hypertension in multivariate analysis. The only associated comorbidities were dyslipidemia (odds ratio: 1.87 [1.24–2.80]) and diabetes (odds ratio: 4.59 [2.43–8.67]).

With respect to diabetes, (S2 Table) increasing age and high BMI, but not sex, were associated with a known diagnosis of diabetes in the multivariate models. Neither the severity of OSA nor nocturia remained significantly associated with diabetes in multivariate analysis. However, a diagnosis of diabetes was significantly associated with hypertension (odds ratio: 4.11 [2.17–7.78]), dyslipidemia (odds ratio: 5.15 [2.89–9.16]) and dysthyroidism (odds ratio: 3.60 [1.72–7.56]).

## Discussion

The study included 633 patients referred to the sleep clinic with suspected OSA. These patients were principally men, with a sex-ratio of almost 4:1, and included a high proportion of patients with severe OSA (43.2%). These findings are consistent with other studies conducted in other countries [12]. Increasing age, male sex and obesity were associated with more severe sleep apnea compared to mild-moderate OSA as measured by the AHI. These constitute the well-characterized ‘triad’ of risk factors for OSA [10, 13, 26].

The majority of referrals for polysomnography were made by chest physicians, which may indicate a lack of awareness on the part of primary care physicians, who were responsible for <5% of referrals, but also of other concerned specialties such as cardiologists, endocrinologists and ENTs. A similar referral pattern has been observed in a previous study of sleep apnea in the Middle East region [18].

The proportion of patients with OSA who were obese (≥30 kg/cm^2^) was over twice as high as the obesity rates in the general Lebanese population [17] and was higher in women than in men. Women with OSA were also older on average than men, which may reflect the increase in prevalence of OSA after menopause. This may indicate the presence of a sex-specific independent risk factor associated with age-related pathophysiological alterations such as estrogen and progesterone depletion [27], and that female sex hormones may have some protective effect on upper airway patency and/or ventilator drive [28].

Most of the patients (97%) were symptomatic with an average of 2–4 symptoms. This finding is not unexpected since patients were generally referred for polysomnography because of these symptoms. This could indicate that asymptomatic patients with sleep apnea could be underdiagnosed. Although the number of symptoms presented was similar in men and women, their nature differed, with snoring and nocturnal choking being more frequent in men, and morning headache and leg discomfort more often reported by women.

Symptoms were generally more frequent in more severe disease although the relationship was statistically significant only for nocturia (OR: 2.35), EDS (OR: 1.89) and witnessed apneas (OR: 1.77). Associations between OSA and both the prevalence and severity of nocturia and OSA have been frequently reported in epidemiological studies [29–31]. Despite the fact that a causal relationship has not been clearly established, some studies have shown that CPAP treatment of OSA decreases nocturia episodes [32]. It has been suggested that the reason why patients with OSA frequently exhibit nocturnal polyuria is that negative intrathoracic pressure swings during apnea leads to atrial stretch and thus the release of atrial natriuretic peptides inducing nocturnal polyuria [31, 33]. Our results suggest that a diagnosis of OSA should be considered in patients reporting nocturia. Although only 17% of patients in our study reported nocturia as a symptom, this is frequently overlooked and underreported by patients and unrecognized by physicians [34], and it is important to ask patients with suspected OSA about this symptom.

Subjective EDS is one of the most frequent symptoms in patients with OSA. It has been demonstrated to be a specific risk factor for hypertension [35–37] and other comorbidities [38]. The prevalence of hypertension has been reported to be higher in patients with OSA complaining of subjective EDS compared to those without such complaints [35–37] and, in this subgroup of patients, CPAP therapy appears to be more effective at controlling and preventing the occurrence of hypertension [37, 39, 40]. In our study, EDS was only associated with arrhythmias and hypertension but not with diabetes or any other comorbidities. The relationship between sleepiness and comorbidities in OSA is likely to be multifactorial, involving increased sympathetic tone, impaired nocturnal autonomic cardiac modulation and metabolic abnormalities, which all contribute to the pathophysiology of OSA [41, 42].

We did not find a sex-related difference in EDS, as reported in certain population-based studies [43]. Several comparisons, from both clinical and population-based studies, report that men more frequently report EDS than women whereas women are more likely than men to complain of atypical symptoms of OSA such as insomnia, headache, fatigue anxiety and depression, [43–45]. In addition, in population-based studies, the ESS is not well correlated with self-reported subjective daytime sleepiness in women compared to men [46]. It is possible that women have a different perception of sleepiness compared with men or that they express this perception differently to men. These atypical symptoms could be a cause of underdiagnosis of OSA in women and to the relatively small numbers of women recruited into sleep studies. [28, 47, 48].

Comorbidities were present in 90% of the patients with OSA with 60% having multiple comorbidities. The presence of multiple chronic diseases, or multimorbidity [49], has been shown to be a predictor of all-cause mortality in OSA patients independently of the severity of OSA [50]. In recent years, several cross-sectional studies have shown an association between OSA and cardiovascular morbidity, particularly hypertension and diabetes. Although these associations appear to be independent of age, weight, and other confounding factors, the evidence emerging from longitudinal studies is to some extent conflicting [39] and the ability of treatment of OSA with CPAP to reduce cardiovascular risk is still debated [40, 51, 52]. In our study, only increasing age and morbid obesity were associated with a self-reported diagnosis of hypertension in the multivariate model. Neither the severity of OSA nor any of the sleep apnea symptoms identified in the univariate analysis remained significantly associated with hypertension. The only associated comorbidities were dyslipidemia and diabetes. With respect to diabetes, only increasing age, high BMI, hypertension, dysthyroidism and dyslipidemia remained significantly associated with a self-reported diagnosis of diabetes in multivariate analysis, a finding suggesting that common risk factors (such as obesity) may underlie the occurrence of these comorbidities and the severity of sleep apnea. However, the cross-sectional design of the present study does not permit any potential causal associations to be investigated.

The study has a number of strengths and limitations. Strengths include the relatively large number of patients enrolled, which provided power for the multivariate regression analyses, and the fact that all patients underwent the same standardized polysomnography procedure, clinical evaluation and careful assessment. The principle limitation of the study was the retrospective nature of the data collection and that a cross-sectional study cannot directly demonstrate causality. This also resulted in some missing data which could not be retrieved. In addition, polysomnography standards, material and rules for interpretation evolved over the seven years of data collection. The use of different criteria for hypopnea (AASM 2007 recommended, AASM 2007 alternative or AASM 2012) may have introduced some heterogeneity in the patient population included. Another limitation is that, after evaluation, most patients were managed by their treating physician outside the sleep center and were not followed by us. For this reason, no information on treatment is available. However, it would clearly be of interest to evaluate treatment of OSA in Lebanon in a future study.

## Conclusions

This is the first study in Lebanon to explore the characteristics of a clinical population of patients with polysomnography-diagnosed OSA. In this cohort of patients, the high proportion of patients whose OSA is severe (43%) and of symptomatic patients (97%) with an average of 2–4 symptoms and the high rate of associated comorbidities could be explained by delays in seeking medical care until their disease reaches a stage where patients are significantly impaired. It may also indicate that asymptomatic patients could be underdiagnosed. The high prevalence of witnessed apnea, nocturia and EDS and their association with the severity of OSA may suggest that these symptoms could be considered as risk predictors for OSA in this population. Women presented more atypical symptoms, which could explain the low representation of women in our cohort and could contribute to the under-evaluation of OSA in women, lower referral rates to sleep clinics and under-representation of women in clinical studies. Given the evidence for clinical under-recognition and low awareness of OSA in the medical community in Lebanon, taking into consideration the referral pattern, a better understanding of sex-specific differences in presentation and of symptom predictors of the risk of OSA, should be promoted both in primary care as well as in concerned medical specialties, to improve diagnosis and management of OSA with a more personalized approach.

## Supporting information

S1 File Study questionnaire—English version. (PDF)

S2 File Study questionnaire—Arabic version. (PDF)

S1 Table Variables associated with hypertension: Multiple logistic regression analysis. (PDF)

S2 Table Variables associated with diabetes: Multiple logistic regression analysis. (PDF)

S1 Data (ZIP)
