# Supplementary material for: Epidemiological characteristics of obstructive sleep apnea in a hospital-based historical cohort in Lebanon
Source: PLoS One. 2020 May 15;15(5):e0231528. doi: 10.1371/journal.pone.0231528 (PMC7228052; doi:10.1371/journal.pone.0231528)
Supplement: S1 File — (PDF) [file pone.0231528.s001.pdf]

S1 File. Study Questionnaire – English Version

SLEEP CLINIC

Dr. M-L COUSSA-KONISKI

GUIDELINES

Please read the questions carefully and answer as they relate to a typical night or your typical sleep pattern. Please consult your bed partner for help in answering the questions, as they may know of problems that may occur during your sleep that you are not aware of.

Please note that:

-Frequently = happens almost every night/day.

-Occasionally = happens one or two times per week.

-Rarely = happens only once in a while.

Answer by circling your choice where appropriate: Do you snore?

Frequently Occasionally Rarely Never

Otherwise write down your answer.

Name: Phone Number:

Age: Profession:

Sexe: F M Date:

1) Describe your sleep problem as best as you can:

____

____

____

2) How long have you had this sleep problem?______________________________

3) Do you snore?

Frequently Occasionally Rarely Never

4) How has your snoring been described?

Loud Moderate Mild

5) How has your snoring been described?

Continuous Intermittent Positional

6) Does your bed partner have to leave the room because of your snoring?

Yes No

7) Do you wake up gasping, wheezing, or short of breath?

Frequently Occasionally Rarely Never

8) Do you feel palpitations during the night?

Frequently Occasionally Rarely Never

9) Do you get chest pain during the night?

Frequently Occasionally Rarely Never

10) Do you toss and turn excessively in your sleep?

Frequently Occasionally Rarely Never

11) Do your legs cramp or jerk upon retiring for sleep or during sleep?

Frequently Occasionally Rarely Never

12) Do you wet your bed at night?

Frequently Occasionally Rarely Never

13) Do you sleep walk?

Frequently Occasionally Rarely Never

14) What is the total duration (in hours) of sleep you get in a typical night's sleep?

____

15) Do you feel rested after a typical night sleep?

Yes No

16) Are you confused and /or disoriented on awakening?

Frequently Occasionally Rarely Never

17) How long does it usually take for you to begin functioning normally when you awaken in the morning?

0-15 min 15-30 min more than 30 min

18) Do you wake up with an irritated or scratchy throat?

Frequently Occasionally Rarely Never

19) Do you wake up with morning headaches?

Frequently Occasionally Rarely Never

20) Do you sleep or get very sleepy whenever you are inactive during the day? (Such as at meetings, watching TV, at movies or in church):

Frequently Occasionally Rarely Never

21) Do you have daytime naps?

Frequently Occasionally Rarely Never

22) Do you have difficultly concentrating or memory problems?

Yes No

23) How many times have you been involved in automobile accidents, as a driver?

____

24) Have you noticed a decrease in your sexual function or interest?

Yes No

25) Have you or any of your family/friends noticed any recent change in your personality?

☞Aggressiveness Yes No

☞Marked irritability Yes No

☞Depression Yes No

26) Do you ever experience muscle paralysis either upon going to sleep or awakening from sleep?

Frequently Occasionally Rarely Never

27) Do you lose muscle control or strength with emotions (anger, laugh, sadness)?

Frequently Occasionally Rarely Never

28) Do you experience vivid dreaming even though you know you are awake?

Frequently Occasionally Rarely Never

29) Does your job require shift work or travel?

Yes No

30) Do you have a history of?

☞ High blood pressure Yes No
☞ Diabetes Yes No
☞ Heart disease Yes No
☞ Breathing problems Yes No
☞ Recent weight gain Yes No

31) Have you had any surgeries in the past?

☞ Tonsillectomy Yes No
☞ Nose surgery Yes No
☞ Thyroid surgery Yes No
☞ Other____

32) How many alcoholic drinks do you take in a usual day?

____

33) How many alcoholic drinks do you usually take before going to bed?

____

34) What sleep medications?

____

____

____

____

35) Do you take antihistaminic? (For allergy, rhinitis………)

Yes No

36) Do any of your family members have the same sleep problem as you?

Yes No
