# Supplementary material for: Epidemiological characteristics of obstructive sleep apnea in a hospital-based historical cohort in Lebanon
Source: PLoS One. 2020 May 15;15(5):e0231528. doi: 10.1371/journal.pone.0231528 (PMC7228052; doi:10.1371/journal.pone.0231528)
Supplement: S2 File — (PDF) [file pone.0231528.s002.pdf]

**S2 File. Study Questionnaire – Arabic Version**

LAUMC
المركز الطبّي للجامعة اللبنانية الأميركية - مستشفى رزق
Lebanese American University Medical Center-Rizk Hospital

## قسم امراض النعاس

## الدكتورة ماري لويز كوسا كونسكي

**ملاحظـــات**

الرجاء قراءة الأسئلة بدقة وإعطاء أجوبة تمثل ليلة نوم عادية بالنسبة لك.

استعين(ي) بزوجتك (بزوجك) للإجابة، فإن لديها (ه) معلومات عن طريقة نومك لا تستطيع أن تعرفها.

**إنتبــه**

- كثير : يحصل تقريبا كل ليلة أو كل يوم
- أحيانا : يحصل مرة أو مرتين في الأسبوع
- نادرا : يحصل مرة من وقت موقت فقط

الرجاء الإجابة بوضع علامة X في المربع المناسب:

□ كثيرا    □ أحيانا    □ نادرا    □ أبدا

أو بكتابة جوابك

الاسم و الشهرة: ________________________ الجنس: □ ذ كر □ انثى

تاريخ الولادة: ____________ الوزن: ____________ الطول:

رقم الهاتف: ____________ المهنة:

قياس العنق: ______________ التاريخ: __________/_________/______________

1) صف مشكلة النوم التي تعاني بأدق ما يمكن:

______________________________________________________

2) منذْ متى تشكو من هذْه المشكلة ؟

______________________________________________________

3) هل تعاني من الشخير ؟

☐ كثيرا ☐ أحيانا ☐ نادرا ☐ أبدا

4) كيف هو الشخير ؟

☐ قوي ☐ متوسط ☐ خفيف

5) هل شخيرك ؟

☐ متواصل ☐ متقطع ☐ حسب طريقة نومك

6) هل يضطر شريكك الى النوم في غرفة أخرى بسبب الشخير ؟

☐ نعم ☐ لا

7) هل تستيقظ وانت تكاد تختنق، مقطوع النفس أو مع صفير في النفس ؟

☐ كثيرا ☐ أحيانا ☐ نادرا ☐ أبدا

8) هل تشعر بدقة قلب سريعة في الليل ؟

☐ كثيرا ☐ أحيانا ☐نادرا ☐ أبدا

9) هل تشعر بألم في صدرك أثناء الليل ؟

☐ كثيرا ☐ أحيانا ☐نادرا ☐ أبدا

10) هل تحرًك رجليك وأنت نائم ؟

☐ كثيرا ☐ أحيانا ☐نادرا ☐ أبدا

11) هل تشعر بتشنُّج في رجليك عندما تستيقظ ؟

☐ كثيرا ☐ أحيانا ☐نادرا ☐ أبدا

12) هل تبوُل في فراشك دون أن تدري في الليل ؟

☐ كثيرا ☐ أحيانا ☐نادرا ☐ أبدا

13) هل تروبص ؟

☐ كثيرا ☐ أحيانا ☐نادرا ☐ أبدا

14) كم ساعة تنام في الليل إجمالا ؟

من __________ساعات الى ______________ ساعات

15) هل تكون مرتاحا عندما تستيقظ صباحا ؟

☐ نعـم ☐ كـلا

16) هل تشعر بأنك مبهم أو مرتبك عندما تستيقظ ؟

☐ كثيرا ☐ أحيانا ☐نادرا ☐ أبدا

17) هل تشعر بألم أو بشحرقة في حنجرتك عندما تستيقظ ؟

☐ كثيرا ☐ أحيانا ☐نادرا ☐ أبدا

18) هل تشعر بصداع في رأسك عندما تستيقظ ؟

☐ كثيرا ☐ أحيانا ☐نادرا ☐ أبدا

19) هل تشعر بالنعاس عندما تكون جالسا في النهار ؟ (أثناء اجتماع، أمام التلفزيون، في السينما أو في الكنيسة)

☐ كثيرا ☐ أحيانا ☐نادرا ☐ أبدا

20) هل تشعر بجاجة للنوم في النهار ؟

☐ نعـم ☐ كـلا

21) هل تشعر بضعف في ذاكرتك أو في قدرتك على التفكير أو التركيز ؟

☐ نعـم ☐ كـلا

22) كم مرُة حصل لك حادث سيارة وأنت السا ئق ؟

______________________________

23) هل تشعر بتدهور في نشا طك الجنسي ؟

☐ نعـم ☐ كـلا

24) هل لاحظت (او احد أصدقائك لاحظ) تغيُر جديد في شخصيتك ؟

-- سريع الغضب : ☐ نعـم ☐ كـلا

-- منهار الأعصاب : ☐ نعـم ☐ كـلا

25) هل تشعر بانشلال عضلاتك عندما تباشر النوم أوعندما تستيقظ ؟

☐ كثيرا ☐ أحيانا ☐نادرا ☐ أبدا

26) هل تحلم وأنت مستيقظ ؟

☐ كثيرا ☐ أحيانا ☐نادرا ☐ أبدا

27) هل بموجب عملك : تعمل ليلا ؟ أو تسافر كثيرا ؟

☐ نعـم ☐ كـلا

28) هل تعاني من :

-الضغط ☐ نعـم ☐ كـلا

-السكري ☐ نعـم ☐ كـلا

-مرض القلب ☐ نعـم ☐ كـلا

-مشاكل في التنفس ☐ نعـم ☐ كـلا

-زيادة جديدة في الوزن ☐ نعـم ☐ كـلا

29) هل خضعت لجراحة في الماضي ؟

جراحة اللوزتين ☐ نعـم ☐ كـلا

جراحة الأنف ☐ نعـم ☐ كـلا

جراحة الغدة (التيرويد) ☐ نعـم ☐ كـلا

غيرها: ________________________________________

30) هل تشرب كحولا ؟

☐ نعـم ☐ كـلا ← عدد الكؤوس: ______________

31) هل تشرب كحولا قبل النوم ؟

☐ نعـم ☐ كـلا

32) هل تستعمل دواءا منوُما أو دواء للأعصاب ؟

☐ نعـم ☐ كـلا ← دوُن أسماءهم : ____________

________________________________________

33) هل تستعمل دواء للحساسية أو الرشح ؟

☐ نعـم ☐ كـلا ← دوُن أسماءهم : __________________

34) هل أحد في عائلتك يشكو من نفس المشكلة أثناء النوم ؟

☐ نعـم ☐ كـلا
