# Supplementary material for: Epidemiological characteristics of obstructive sleep apnea in a hospital-based historical cohort in Lebanon
Source: PLoS One. 2020 May 15;15(5):e0231528. doi: 10.1371/journal.pone.0231528 (PMC7228052; doi:10.1371/journal.pone.0231528)
Supplement: S1 Table — (PDF) [file pone.0231528.s003.pdf]

S1 Table. Variables associated with hypertension: multiple logistic regression analysis

Variable	Reference	Univariate *p*	Multivariate *p*	Multivariate OR [95% CI]
Age	≥70 years vs <70 years	<0.001	0.022	**1.71 [1.08 – 2.72]**
Sex	Male vs female	0.402	0.556	0.85 [0.48 – 1.48]
BMI	≥30 kg/m^2 vs <30 kg/m^2	<0.001	0.015	**1.71 [1.11 – 2.64]**
Snoring	Present vs absent	0.020	0.344	1.27 [0.77 – 2.10]
EDS	Present vs absent	0.011	0.691	1.10 [0.70 – 1.72]
Nocturia	Present vs absent	0.001	0.216	1.40 [0.82 – 2.40]
OSA	Severe vs moderate/mild	<0.001	0.080	0.69 [0.45 – 1.05]
Dyslipidemia	Present vs absent	<0.001	0.003	**1.87 [1.24 – 2.80]**
Diabetes	Present vs absent	<0.001	<0.001	**4.59 [2. 43 – 8.67]**
Arrhythmias	Present vs absent	<0.001	0.255	1.50 [0.75 – 3.00]
CHD	Present vs absent	0.016	0.745	1.19 [0.42 – 3.40]
Dysthyroidism	Present vs absent	0.016	0.592	0.82 [0.40 – 1.68]
MSK disorders	Present vs absent	0.025	0.560	0.79 [0.37 – 1.72]

BMI: body mass index; EDS: excessive daytime sleepiness; OSA: obstructive sleep apnea; MSK: musculoskeletal.
