# Supplementary material for: Epidemiological characteristics of obstructive sleep apnea in a hospital-based historical cohort in Lebanon
Source: PLoS One. 2020 May 15;15(5):e0231528. doi: 10.1371/journal.pone.0231528 (PMC7228052; doi:10.1371/journal.pone.0231528)
Supplement: S2 Table — (PDF) [file pone.0231528.s004.pdf]

S2 Table. Variables associated with diabetes: multiple logistic regression analysis

Variable	Reference	Univariate *p*	Multivariate *p*	Multivariate OR [95% CI]
Age	≥70 years vs <70 years	<0.001	0.027	**1.85 [1.07 – 3.18]**
Sex	Male vs female	0.001	0.239	0.68 [0.36 – 1.29]
BMI	≥30 kg/m^2 vs <30 kg/m^2	<0.001	0.003	**2.67 [1.39 – 5.13]**
Nocturia	Present vs absent	0.013	0.589	1.19 [0.63 – 2.27]
OSA	Severe vs moderate/mild	0.010	0.447	0.81 [0.47 – 1.40]
Hypertension	Present vs absent	<0.001	<0.001	**4.11 [2.17 – 7.78]**
Dyslipidemia	Present vs absent	<0.001	<0.001	**5.15 [2.89 – 9.16]**
CHD	Present vs absent	0.001	0.312	1.70 [0.61 – 4.78]
Dysthyroidism	Present vs absent	<0.001	0.001	**3.60 [1.72 – 7.56]**
GERD	Present vs absent	0.038	0.151	0.68 [0.40 – 1.15]

BMI: body mass index; CHD: coronary heart disease; OSA: obstructive sleep apnea; GERD: gastro-esophageal reflux disorder.
